# Identification of Key Contributory Factors Responsible for Vascular Dysfunction in Idiopathic Recurrent Spontaneous Miscarriage

**DOI:** 10.1371/journal.pone.0080940

**Published:** 2013-11-15

**Authors:** Priyanka Banerjee, Sanghamitra Ghosh, Mainak Dutta, Elavarasan Subramani, Jaydeep Khalpada, Sourav RoyChoudhury, Baidyanath Chakravarty, Koel Chaudhury

**Affiliations:** 1 School of Medical Science and Technology, Indian Institute of Technology, Kharagpur, India; 2 Institute of Reproductive Medicine, Kolkata, India; CHA University, Republic of Korea

## Abstract

Poor endometrial perfusion during implantation window is reported to be one of the possible causes of idiopathic recurrent spontaneous miscarriage (IRSM). We have tested the hypothesis that certain angiogenic and vasoactive factors are associated with vascular dysfunction during implantation window in IRSM and, therefore, could play a contributory role in making the endometrium unreceptive in these women. This is a prospective case-controlled study carried out on 66 women with IRSM and age and BMI matched 50 fertile women serving as controls. Endometrial expression of pro-inflammatory (IL-1β, TNF-α, IFN-γ, TGF-β1), anti-inflammatory (IL-4, -10), angiogenesis-associated cytokines (IL-2, -6, -8), angiogenic and vasoactive factors including prostaglandin E2 (PGE2), vascular endothelial growth factor (VEGF), endothelial nitric oxide synthase (eNOS), nitric oxide (NO) and adrenomedullin (ADM) were measured during implantation window by ELISA. Subendometrial blood flow (SEBF) was assessed by color Doppler ultrasonography. Multivariate analysis was used to identify the significant factor(s) responsible for vascular dysfunction in IRSM women during window of implantation and further correlated with vascular dysfunction. Endometrial expression of pro-inflammatory cytokines and PGE2 were up-regulated and anti-inflammatory and angiogenesis-associated cytokines down-regulated in IRSM women as compared with controls. Further, the angiogenic and vasoactive factors including VEGF, eNOS, NO and ADM were found to be down-regulated and SEBF grossly affected in these women. Multivariate analysis identified IL-10, followed by VEGF and eNOS as the major factors contributing towards vascular dysfunction in IRSM women. Moreover, these factors strongly correlated with blood flow impairment. This study provides an understanding that IL-10, VEGF and eNOS are the principal key components having a contributory role in endometrial vascular dysfunction in women with IRSM. Down-regulation of these factors is also associated with impaired endometrial perfusion which possibly makes the endometrium unreceptive that may eventually cause early pregnancy loss.

## Introduction

Recurrent spontaneous miscarriage (RSM), affecting 1-3% of fertile couples, is defined as spontaneous abortion of three or more clinically diagnosed pregnancies within less than 24 weeks of gestation [1]. Various causative factors such as genetic, endocrine, anatomic, immunological, infectious, environmental, thrombophilic and metabolic are thought to be responsible for RSM. Regardless of extensive research undertaken in this field, etiology of ~50% of these cases still remains unknown and hence poses to be a clinical challenge [2]. An unreceptive endometrium, leading to abnormal implantation, is believed to be associated with idiopathic recurrent spontaneous miscarriage (IRSM). However, whether the relationship is a causal or casual one remains to be established [3].

It is evidenced that blood flow through the uterine arteries is partly dependent on various angiogenic and vasoactive factors [4]. A good blood supply towards the endometrium is usually considered to be a marker for endometrial receptivity [5]. A decrease in sub-endometrial blood flow (SEBF) is associated with reduced pregnancy rate [6]. Platelet endothelial cell adhesion molecule (PECAM-1), a transmembrane glycoprotein, is a useful marker of blood vessel formation. It expresses constitutively on all vascular cells and plays a crucial role in angiogenesis. Reduced expression of PECAM-1 is reported in cytotrophoblasts, syncytiotrophoblasts, and extravillous trophoblasts of the abortion materials in women with spontaneous abortion [7]. The endothelium of uterine blood vessels uses NO, produced in the blood vessel by endothelial nitric oxide synthase (eNOS), to signal the surrounding smooth muscles to relax. This results in vasodilation, thereby making the endometrium receptive [8]. Further, increased expression of endometrial eNOS during mid-secretory phase suggests its role in endometrial receptivity and blastocyst implantation [9]. Adrenomedullin (ADM) is another vasoactive peptide which has drawn considerable attention in recent years in view of its involvement in regulating the expression of pinopodes which facilitate endometrium-blastocyst attachment [10]. The influence of these factors on SEBF during implantation window in women with IRSM is not reported so far. 

This study is a continuation of our previous work where an aberrant expression of inflammatory and angiogenic factors concomitant with down regulation of anti-inflammatory and angiogenesis-associated cytokines was observed during implantation window in women with IRSM [11]. The expression of vasodilators including ADM, eNOS and NO during implantation period of women with IRSM is investigated in the present study. Pro-inflammatory, anti-inflammatory and angiogenesis-associated cytokines and angiogenic factors are included and taken together, multivariate data analysis applied to identify the significant factor(s) responsible for vascular dysfunction in IRSM women during window of implantation. Further, SEBF is estimated in these women and correlated with the major factor(s) identified.

## Materials and Methods

### Patient selection

The present study was conducted at the Institute of Reproductive Medicine, Kolkata India, a tertiary care hospital and School of Medical Science and Technology, Indian Institute of Technology, Kharagpur, India. 

100 women (age<35 years, BMI≤28), who have had three or more consecutive miscarriages within the first trimester (upto 12 weeks of gestation) with no apparent cause, reporting at the Institute of Reproductive Medicine, for infertility treatment were initially identified for the study (IRSM group). The women were not associated with any other gynecological disorder, and had not received any kind of medication since the last three months. All women included in the study were south Asian of Indian origin.

The following tests were performed to confirm that there was no apparent cause of recurrent pregnancy loss: TSH and anti-thyroid antibody tests, antiphospholipid antibodies tests (anticardiolipin antibodies and Lupus anticoagulants IgG and IgM), TORCH (Toxoplasmosis, Rubella, Cytomegalovirus and Herpes) tests, paternal and maternal chromosomal analysis, hysterosalpingography and hysteroscopy to rule out uterine defects, abnormal fasting level of homocysteine, PCOS, exclusion of diabetes mellitus, and estimation of mid-luteal serum progesterone to exclude luteal phase defect. 

Women suffering from recurrent miscarriage due to any other pre-existing medical conditions like antiphospholipid syndrome, hormonal disorder, chromosomal defects, thrombophilia, hyper-homocystinemia, infectious diseases, PCOS, uterine malformation, environmental causes, gestational hypertension and other known causes were excluded. Further, women with male partners having fragmented sperm DNA, sperm meiotic alternations and poor sperm parameters were also excluded from the study. 100 proven fertile women (age and BMI-matched) undergoing sterilization were identified as controls. Women of these groups had parity between 2-5 (2.5±0.12) and normal regular menstrual cycles. They had no history of failed pregnancies and other significant clinical abnormalities. 

Out of the total 200 women, 84 subjects had to be excluded; 34 from the IRSM group and 50 from controls. These women either did not report for (a) serial folliculometry to confirm ovulation during the specified period or for (b) endometrial biopsy collection during day 18-22 of their menstrual cycle (implantation window) or the (c) endometrial tissue collected from several women was highly flaky or inadequate, rendering these samples unsuitable for the experimental study or (d) did not express interest in participating in the study. Power calculation was subsequently performed on the reduced sample size of 66 IRSM women and 50 controls cases. A power > 99% for this sample size at a significance level α of 0.05 on comparing both the groups was observed. 

### Ethics statement

The study was approved by the Institute Ethics Committee, Institute of Reproductive Medicine (IRM/IEC/BNC-IHP-35/26-02-2010) and written informed consent obtained from all couples.

### Sample collection

Ultrasonography (USG) for serial folliculometry was performed day 10 onwards in all cases to monitor follicular growth till ovulation occurred. Following confirmation of ovulation, endometrial biopsy was obtained from all women of both the groups during day 18-22 of their menstrual cycle under general anaesthesia by dilation and curettage. The endometrial biopsies were sent for routine pathologic analysis where endometrial histological dating was performed according to Noyes criteria. After washing with phosphate buffer saline (PBS), a part of the collected tissue was fixed for immunohistochemistry (IHC). The other part was weighed and ~50 mg of tissue homogenized using a tissue grinder in a 3 ml of tissue extraction buffer (0.5 M Tris–HCl pH 7.6, 0.2 M NaCl, 10 mM CaCl_2_ and 1% (w/v) Triton X-100). Volumes of extraction buffer were determined relative to the amount of tissue present. Tissue homogenate was then incubated at 4°C for 45 min under gentle shaking and centrifuged at 12 000 *g* for 30 min at 4°C to recover the soluble extract. The protein concentration of the homogenates was determined using GeNei™ Protein Estimation Kits (Bangalore Genei, India). Final protein concentration of the tissue homogenate was >1 mg/ml. The homogenate was then stored at -80°C, until further use.

### In vitro measurements

Our earlier report in 36 IRSM cases and 30 controls where endometrial expression of various pro-inflammatory, anti-inflammatory, angiogenesis-associated cytokines, and angiogenic factors were compared [11], is extended to a larger sample size in the present study. Thirty additional cases of IRSM and 20 controls are included. In addition to interleukin (IL)-1β, tumor necrosis factor (TNF)-α, interferon (IFN)-γ, transforming growth factor (TGF)-β1, IL-4, IL-10, IL-2, 6, 8, vascular endothelial growth factor (VEGF), and prostaglandin (PG) E2 assessment, endothelial nitric oxide synthase (eNOS), nitric oxide (NO), adrenomedullin (ADM) and SEBF are estimated during implantation window in these women. Serum estradiol and progesterone were also measured during the day of tissue collection.

### ELISA

Tissue homogenates containing protein concentration of 30 μg/ml were used for estimating the level of IL-1β, TNF-α, IFN-γ, TGF-β1, IL-10, -2, -6, -8, VEGF, eNOS and ADM. VEGF and PGE2 were assessed using anti-human rabbit polyclonal antibody to VEGF (ab9570) and PGE2 (ab-2318; Abcam, Cambridge, UK), respectively employing quantitative direct enzyme immunoassay technique. IL-1β, TNF-α, IFN-γ, IL-10, -4, -2, -6, and -8 were measured using human IL-1β (557966), TNF-α (550610), IFN-γ (550612), IL-10 (550613), IL-4 (550614), IL-2 (550611), IL-6 (550799), and IL-8 (550999) ELISA Kit (BD Biosciences, San Jose, CA, USA), respectively. TGF-β1 was analysed using anti-human mouse monoclonal antibody to TGF-β1 (sc-57443; Santacruz, Inc. USA). eNOS and ADM were analysed using anti-human rabbit polyclonal antibody to eNOS (sc-8311) and ADM (sc-33187; Santacruz, Inc. USA), respectively. The principle of the assay is based on the quantitative sandwich enzyme immunoassay technique. The intra- and inter-assay variability was respectively 2.5% and 3.11% for IL-1β, 2.7% and 5.3% for TNF-α, 3.2% and 4.3% for IFN-γ, <7 and <8% for TGF-β1, 3.7% and 2.6% for IL-4, 2.1% and 5.8% for IL-10, 3.1% and 3.3% for IL-2, 4.1% and 7.9% for IL-6, 4% and 3.2% for IL-8, 5.3% and 7.6% for VEGF, <5% and <14% for ADM, 4.4% and 9.2% for PGE2, and 8% and 10% for eNOS. Samples with expression level less than the limit of detection were assigned as half of the sensitivity limit.

### Nitric oxide (NO) measurement

NO was measured in the tissue as nitrite/nitrate, by the Griess reaction [12]. 100 µl of tissue homogenate sample was mixed with an equal volume of Griess reagent (0.1% (1-naphthyl) ethylene diamine dihydrochloride, 1% sulfanilamide, and 2.5% phosphoric acid) and the resulting absorbance measured at 550 nm in a microplate reader. Background (blank) was determined in each experiment by incubating the reagent without samples. Amount of NO in each sample was determined using a standard curve generated with known concentration of NO and its concentration expressed as µM/l. The inter- and intra-assay viability co-efficient was 4.1% and 2.9%, respectively.

### SEBF measurement

The endometrial thickness, together with SEBF were evaluated immediately before endometrial curettage during the window of implantation. This was done in both the groups using transvaginal color Doppler and two dimensional Power Doppler using a 7.5 MHz probe (Medison SA 9900, South Korea). The resistance index {RI = (peak systolic velocity – end diastolic velocity)/peak systolic velocity}, pulsatility index {PI = (peak systolic velocity – end diastolic velocity)/mean velocity}, and systolic/diastolic ratio (S/D ratio = systolic velocity / end diastolic velocity) were calculated on three consecutive waveforms. USG was performed in all patients by only one person to avoid inter-observer variation and the sonologist blinded to avoid significant bias. For assessing the reproducibility of Doppler measurements, RI, PI and S/D ratio were measured in 10 patients by the same operator 3 times at 10-minute intervals and the coefficient of variation was found to be 5.8%.

### Immunohistochemistry

3-5 µm thick sections obtained from formaldehyde fixed, paraffin-embedded tissue were dehydrated in graded ethanol. After antigen retrieval, slides were incubated for an hour in 3% blocking serum (BSA) in PBS for controlling non-specific binding of primary antibody. The slides were then incubated with goat anti-PECAM 1 (sc-1506; Santacruz biotechnology, INC., Santa Cruz, California, USA). Excess primary antibody was washed with PBS and the sections were again incubated with anti-goat (ab6885) secondary antibody (Abcam, Cambridge, UK) according to the manufacturer’s protocol. Labeled cells were visualized with diaminobenzidine (DAB) and sections counterstained with hematoxylin. Next, the slides were dehydrated using series of alcohol gradient and mounted using distrene, tricresyl phosphate (DPX) and xylene. The slides were then examined under bright field microscope (Carl Zeiss, Jena, Germany). The DAB staining intensities on each slide were individually graded by visual inspection. Semi-quantitative scoring was done independently by two observers to assess the staining intensity which provides a measure of the expression of these molecules. Immunostaining was classified on the basis of intensity of staining ((no staining-0; weak-1 point; moderate-2 points; strong-3 points) and percentage/extent of stained cells (0%-0 point; <10%-1 point; 11%-50%-2 points; 51%-80%-3 points; >80%-4 points). A final immunohistochemical score (Score 0-12) were obtained by multiplying intensity score and extent of stained cells.

### Statistical Analysis

To estimate relationships between different variables, and correlate how important each one is to the final outcome where dependencies exist, multivariate analysis was performed for dimension reduction of variables. A part of the data used here for multivariate analysis originates from the previous study [10]. Data matrix was created with samples in rows and variables (experimentally determined factors) in columns. Normalization (by log transformation) was performed in order to minimize possible differences in concentration between samples. After data pre-processing, principal component analysis (PCA) was performed using SIMCA 13.0.2. PCA is an unsupervised multivariate projection method designed to extract and display systematic variations within the data matrix. It is primarily used to detect intrinsic clusters and outlier (observations that are extreme or that do not fit the PCA model) within the data set. Followed by sample clustering using PCA, partial least squares discriminant analysis (PLS-DA) analysis was performed using SIMCA 13.0.2. PLS-DA is another method of multivariate statistical analysis which is used for constructing predictive models. Unlike PCA, PLS-DA is a robust form of analysis, directed towards factor space that are associated with high variation in the responses but biased towards directions that are accurately predicted (in this case, sample groups i.e. diseased and controls). 

Statistical significance was defined as p≤0.05. Pearson linear correlation was used to identify those angiogenic and vasculogenic factors which correlate highly with the Doppler parameters. Ky Plot version 2.0 beta 13 software was used for this purpose.

## Results

**Figure 1:**
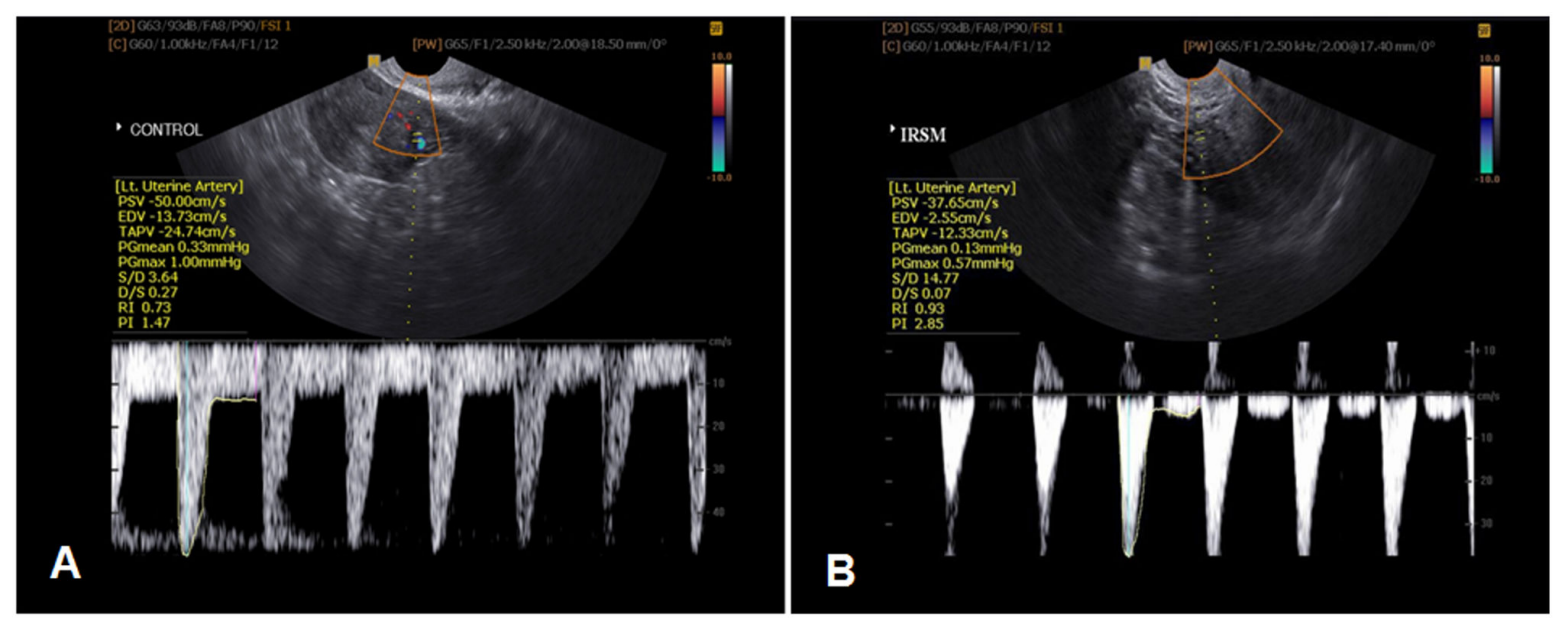
Typical 2D Doppler ultrasound image of sub-endometrial blood flow measured in (A) controls and (B) IRSM.

The demographic characteristics are summarized in Table 1. No significant differences were observed in terms of age, BMI, endometrial thickness, serum estrogen and progesterone levels between women with IRSM and controls. USG of the endometrium indicated PI, EDV and S/D ratio to be grossly affected in women with IRSM as compared with controls. RI, however, was found to be comparable between the two groups (Table 2; Figure 1). 

**Table 1:** Clinical characteristics of women with idiopathic recurrent spontaneous miscarriage (IRSM) and controls during implantation window.

**Parameters**	**IRSM**	**Controls**	**P value**
**Age**	28.8±3.1	28.56±2.92	NS
**BMI**	27.82±1.55	27.26±1.92	NS
**Endometrial thickness (mm)**	8.71±1.55	9.11±1.68	NS
**Serum estrogen level (pg/ml)**	227.03±60.4	205.04±61.8	NS
**Serum progesterone level (ng/ml)**	18.35±3.58	19.25±3.02	NS

Unpaired Student's t-test is used to compare the differences between parametric data sets.

**Table 2:** Doppler indices for sub-endometrial blood flow in women with idiopathic recurrent spontaneous miscarriage (IRSM) and controls during implantation window.

**Uterine Artery Doppler Indices**	**IRSM**	**Controls**	**P Value**
**Resistivity Index**	0.88 ± 0.1	0.85 ± 0.15	NS
**Pulsatility Index**	2.09 ± 0.61	1.18 ± 0.4	P<0.05
**End-Diastolic Velocity (cm/sec)**	4.32 ± 1.81	12.17 ± 2.71	P<0.05
**Systolic/Diastolic (S/D) ratio**	20.23 ± 3.9	2.36 ± 0.74	P<0.05

Unpaired Student's t-test is used to compare the differences between parametric data sets.

**Figure 2:**
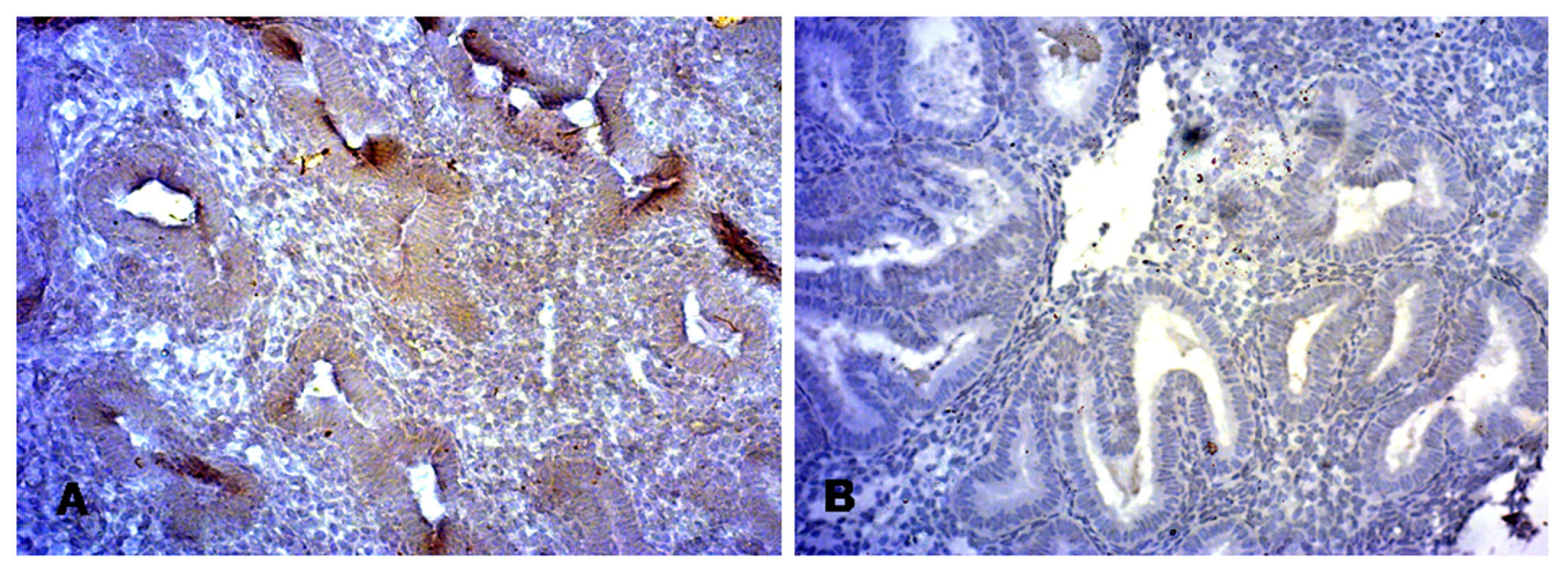
Immunohistologic images of expression of endometrial PECAM-1/CD31 in (A) controls and (B) IRSM.

Levels of the vasoactive factors including eNOS, NO and ADM estimated during implantation window were found to be significantly less in IRSM women as compared with controls. The angiogenic factors, pro-inflammatory, anti-inflammatory and angiogenesis-associated cytokines showed a similar trend as reported in our previous study on a smaller sample size. IL-1β, TNF-α, IFN-γ, TGF-β1 and PGE2 were found to be up-regulated and IL-4, IL-10, IL-2, 6, 8 and VEGF down-regulated in IRSM cases as compared with controls (Table 3). PECAM-1, the marker of angiogenesis and vascular development, exhibited low immunoreactivity levels in women with IRSM in contrast to strong immunoreactivity observed in controls (Table 4; Figure 2). 

**Table 3:** Expression levels of endometrial pro-inflammatory, anti-inflammatory, angiogenesis associated cytokines, angiogenic and vasoactive factors during the implantation window period in women with idiopathic recurrent spontaneous miscarriage (IRSM) and controls as determined by parametric t-test for unpaired comparison.

**Parameters**	**IRSM**	**Controls**	**P value**
**IL-1β (pg/ml)**	35.17±6.02	9.28±2.47	P<0.05
**TNF-α (pg/ml)**	34.91±4.88	12.01±4.51	P<0.05
**IFN-γ (pg/ml)**	26.36±4.91	5.62±2.32	P≤0.05
**TGF-β1 (pg/ml)**	167.54±40.05	96.05±23	P<0.05
**IL-4 (pg/ml)**	27.74±6.7	103.49±15.53	P<0.05
**IL-10 (pg/ml)**	28.07± 4.77	95.44± 8.29	P<0.05
**IL-2 (pg/ml)**	13.86±4.64	44.66±5.35	P<.05
**IL-6 (pg/ml)**	2.4±0.94	15.29±2.84	P<0.05
**IL-8 (pg/ml)**	74.37±8.69	130.96±16.3	P<0.05
**VEGF (pg/ml)**	121.62± 14.93	315.04± 30.42	P<0.05
**PG E2 (pg/ml)**	835.22± 174.16	510.38± 155.34	P<0.05
**Adrenomedullin (pg/ml)**	943.4±61.37	1156.2 ± 52.91	P<0.05
**NO (µM/l)**	8.38±1.39	12.72±1.42	P<0.05
**eNOS (pg/ml)**	852.73±45.17	1259.24±52.02	P<0.05

**Table 4:** Scoring method based on expression patterns of PECAM-1 markers in endometrium during implantation window of women with IRSM and controls by immunohistochemistry.

**Marker**	**Scoring Categories**	**IRSM**	**Control**	**p value**
PECAM-1	0-2	50	8	P<0.0001
	3-12	16	42	

**Figure 3:**
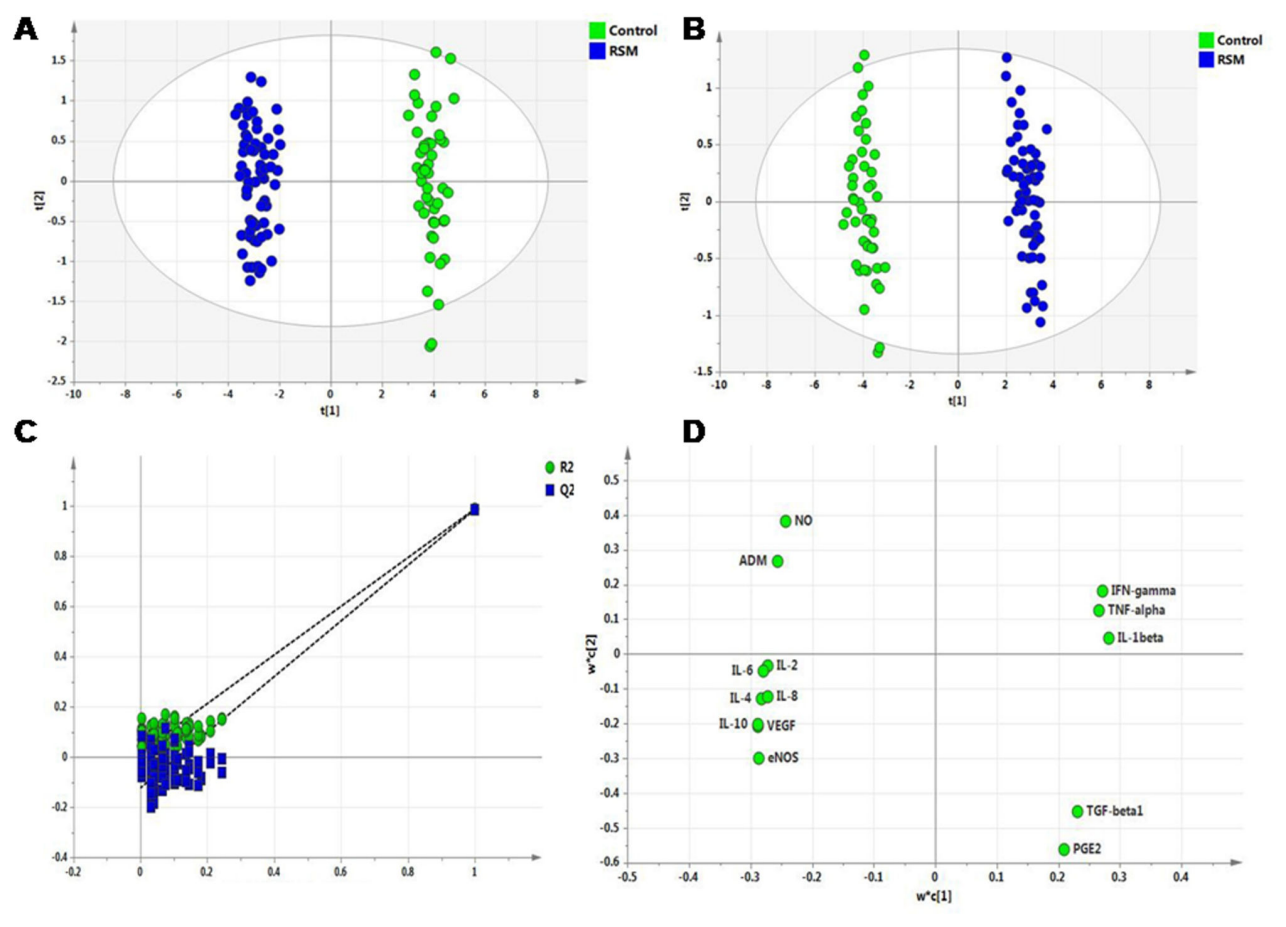
Multivariate data analyses of factors responsible for vascular dysfunction. (A) Scores scatter plot t1 vs. t2 resulting after applying PCA to endometrial expression of various angiogenic and vasoactive factors of controls (green) and IRSM (blue), (B) scores scatter plot t1 vs t2 resulting after applying PLS-DA to endometrial expression of various angiogenic and vasoactive factors of controls (green) and IRSM (blue), (C) Results from the permutation test for the IRSM group suggest a valid PLS-DA model. The vertical axis is the R2 and Q2 values of each model and the horizontal axis shows the correlation between the permuted class vectors and the original class vector. The original class has the correlation 1.0 with itself, defining the high point on the horizontal axis. All R2 and Q2 values calculated from the permuted data are lower than the original model in the validation plot. Y-axis intercepts: R2= (0.0, 0.0189), Q2= (0.0, −0.123). (D) Loading scatter plot indicates factors IL-1β, TNF-α, IFN-γ, TGF-β1, PGE2 are upregulated in IRSM and factors IL-2, IL-4, IL-6, IL-8, IL-10, VEGF, ADM, eNOS, NO are upregulated in controls.

Exploratory PCA was employed to detect intrinsic clustering and possible outliers. Scatter plot of t1 vs t2 indicates unsupervised (unbiased and having no prior knowledge of sample groups) separation trend between IRSM and controls (Figure 3A). The group separation was further maximized by PLS-DA (Figure 3B). The predictive capability (Q2) and explained variance (R2) were extracted for the PLS-DA model. The model with both R2 and Q2 well above 0.9 indicated a very good predictive ability. Further, validation of the PLS-DA model was performed using permutation test statistics. This validation is performed to compare the R2 and Q2 of the original model with the R2 and Q2 of several models based on data where the order of the class variable is randomly permuted, while the other data matrix is kept intact. Results of permutation test statistics indicated the original model to be far more superior than all the one hundred model generated by random permutation of class variable (Figure 3C). 

**Figure 4:**
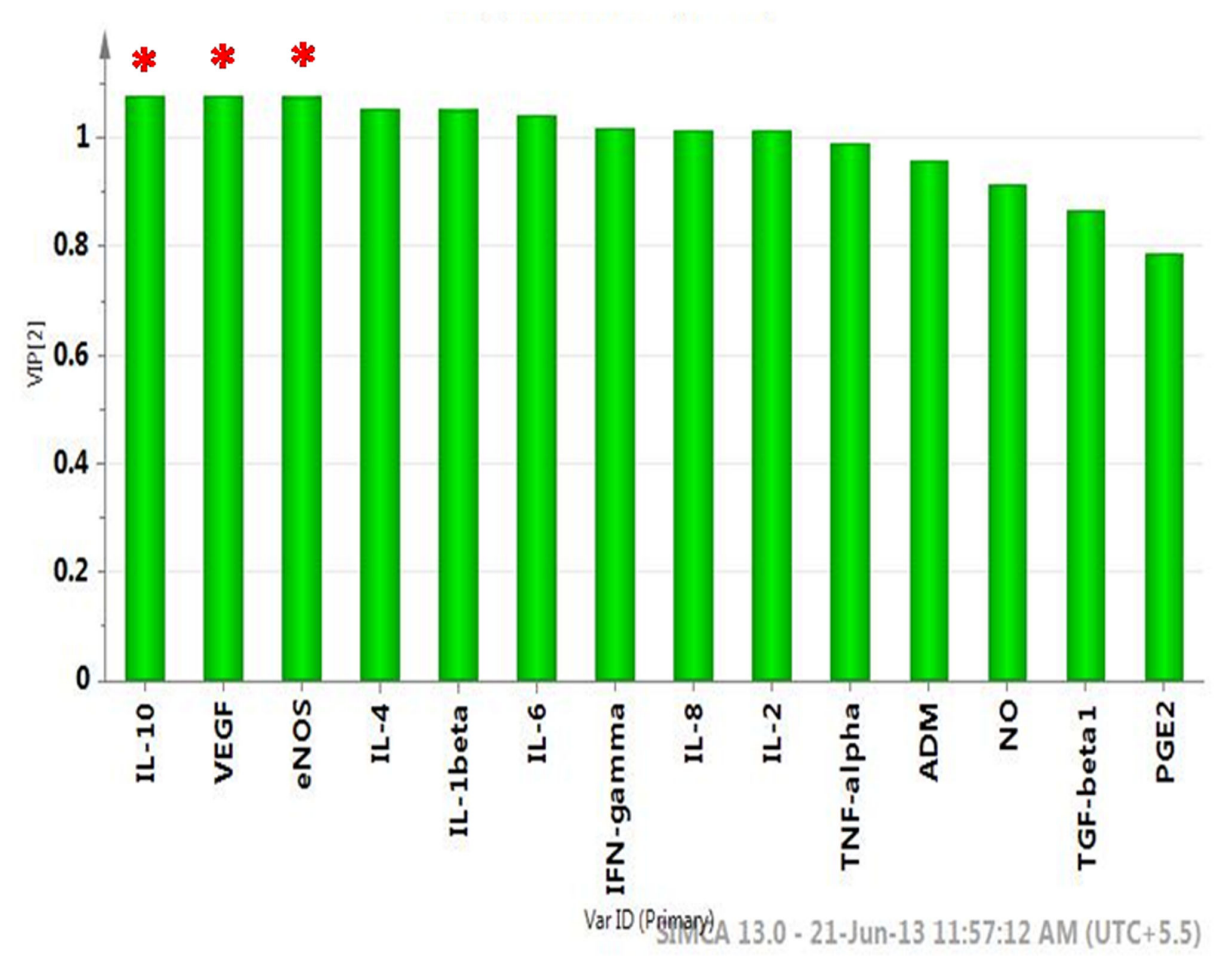
Important features identified by PLS-DA and VIP scores. A VIP score is a measure of a variable’s importance in the PLS-DA model. It summarizes the contribution a variable makes to the model. The VIP score of a variable is calculated as a weighted sum of the squared correlations between the PLS-DA components and the original variable. The weights correspond to the percentage variation explained by the PLS-DA component in the model. The number of terms in the sum depends on the number of PLS-DA components found to be significant in distinguishing the classes. The Y axis indicates the VIP scores corresponding to each variable on the X- axis. The red asterisks indicate the factors with the highest VIP scores and thus are the most contributory variables in class discrimination in the PLS-DA model.

Factors with higher loadings were identified (Figure 3D). Further a variable lies away from the plot origin, the stronger impact the variable has on the model. Comparing the loading and scores plot, pro-inflammatory cytokines (IL-1β, TNF-α, IFN-γ, TGF-β1) and PGE2 showed positive factor 1 loading, hence indicating more bias towards IRSM. Similarly, angiogenic and vasoactive factors (VEGF, NO, ADM, eNOS), anti-inflammatory (IL-4 and IL-10) and angiogenesis-associated cytokines (IL-2, 6, 8) showed negative factor 1 loading, indicating bias towards the control group. Factors with high variable importance in projection (VIP) scores are regarded as significant and, therefore, considered for quantitative analysis of variation. IL-10 followed by VEGF and eNOS were identified as the prime factors corresponding to three highest VIP scores (Figure 4). Further, IL-10, VEGF and eNOS, the three major regulatory factors identified, showed significant correlation with the Doppler indices. A significant positive correlation was observed between IL-10 and PI (r = 0.79, P < .001), VEGF with EDV (r = 0.61, P < .001), and eNOS with EDV (r = 0.74, P < .001). The S/D ratio showed a significant negative correlation between VEGF (r = -0.78, P < .001) and eNOS (r = -0.67, P < .001), respectively.

## Discussion

A hypothesis that certain angiogenic and vasoactive factors are associated with vascular dysfunction and could play a contributory role in poor endometrial receptivity in IRSM is proposed. To ascertain this hypothesis, the major factors responsible for angiogenesis and vascularisation and expressed during implantation window in women with IRSM were estimated. Further, multivariate analysis including PCA and PLS-DA was used to determine the key components that should be retained for further analysis. 

On plotting t1 × t2, the plots for IRSM are observed to lie in a principal component plane which is significantly different from the plane where plots for controls are clustered (Figure 3A) PLS-DA indicates statistically significant separation between IRSM and control cases (Figure 3B). A quantitative measure of the goodness of fit in the PLS-DA model is given by the parameter R2, which varies between 0 and 1; with 1 representing a perfect fit and 0, no fit at all. However R2 is inflationary and approaches unity as model complexity increases; model complexity is governed by model parameters and number of components. It is, therefore, not sufficient to have a high R2 value. On the other hand, the goodness of prediction represented by Q2 is less inflationary and does not automatically come close to 1 with increasing model complexity. In short, both R2 and Q2 are important to assess the validity of the model. Our values corresponding to R2 near to 0.989 and Q2 close to 0.986 indicate that the model is valid and can predict better than chance. Another validation, showing that the PLS-DA model not only just fits the training set well but also predicts class well for new observations, was performed using permutation test statistics. The permutation test assured the validity of the PLS-DA model with all R2 and Q2 values calculated from the permuted data lower than the original one in the validation plot. Q2 intercepted the y-axis at −0.123 (Figure 3C). VIP scores indicate the importance of each variable in the projection used in a PLS-DA model and is often used for variable selection. Variables with top three VIP scores, IL-10, VEGF and eNOS were identified as the three prime factors contributing to vascular dysfunction in the IRSM group. 

The importance of IL-10, VEGF and eNOS in implantation is well established. IL-10, a potent vascular cytokine, is reported to inhibit inflammation-mediated vascular dysfunction [13]. Several studies have associated IL-10 deficiency to recurrent spontaneous abortion [14,15]; however, the mechanisms that may lead to poor IL-10 production at the maternal–fetal interface are not well understood. Moreover, there are no studies reported so far on the endometrial expression of IL-10 during implantation window in women with IRSM. A significant decrease was seen in the level of this cytokine during peri-implantation period in IRSM women as compared to controls. This is supported by the earlier findings of Bates et al. (2002) where spontaneous abortions have been correlated with decreased production of IL-10 by peripheral blood mononuclear cells as compared with normal pregnancy [16].

The angiogenic factor, VEGF plays an important role during implantation by stimulating endothelial cell proliferation and increasing vascular permeability [17]. A significant increase in VEGF during implantation period in normal fertile women is documented [18,19]. We observed a significant decrease in the expression of endometrial VEGF in women with IRSM. This finding is attributed to the reduced expression of angiogenesis-associated cytokines in IRSM which interferes with the process of angiogenesis during the preparation of a receptive endometrium [11]. 

Expression of endometrial eNOS protein and mRNA is known to vary during the menstrual cycle of normal fertile women, with maximal expression during the mid-secretory phase [20]. It is well established that lack of eNOS-derived nitric oxide is associated with vasospasm and vascular infarction in IRSM [21,22]. In addition, eNOS gene polymorphism has been found in these women and is suggested to be a genetic determinant of the risk of IRSM [22,23]. This is also reflected in our present findings where down-regulated eNOS emerges to be one of the key factors playing a contributory role in IRSM. A correlation between eNOS and NO is also observed (r = 0.53, P < .001). It is hypothesized that reduced eNOS and NO expression observed in IRSM women is possibly due to associated eNOS polymorphism. Contrary to our findings, there is only one report by Najafi et al. (2012) where a significantly higher endometrial eNOS expression in women with recurrent miscarriage is reported [9]. The group hypothesizes that excessive expression of eNOS causes excess NO generation, which leads to failure of early pregnancy maintenance. Although interesting, these findings need further validation in view of the fact that the sample size is rather small (n=10). 

Poor endometrial perfusion is reported to be a possible cause of IRSM [24]. It is likely that the significant decrease in the expression of IL-10, VEGF and eNOS in IRSM women is associated with vascular dysfunction and poor endometrial receptivity. This possibility of impairment in uterine perfusion motivated us to explore whether a relationship exists between these three major vasoactive factors and SEBF. A high correlation was observed between IL-10 and PI (r = 0.79, P < .001). VEGF showed significant positive correlation with EDV (r = 0.61, P < .001) and significant negative correlation with S/D ratio (r = -0.78, P < .001). eNOS showed a similar trend, correlating significantly with EDV (r = 0.74, P < .001) and negatively with S/D ratio (r = -0.67, P < .001). Additionally, we observed reduced endometrial expression of PECAM-1 in IRSM which indicates defect in endometrial vascular development and angiogenesis (Figure 2). It is evident from these findings that there exists an association between impaired uterine blood flow and angiogenesis and vascularisation-associated factors during peri-implantation period in IRSM women. 

Summarizing, IL-10, VEGF and eNOS emerge to be the principal key components having a contributory role in endometrial vascular function of women with IRSM. Down-regulation of these vasoactive factors during implantation window appears to cause dysfunction in uterine perfusion, resulting in an unreceptive endometrium that may eventually cause early pregnancy loss. 
